# YY1 Promotes Endothelial Cell-Dependent Tumor Angiogenesis in Hepatocellular Carcinoma by Transcriptionally Activating VEGFA

**DOI:** 10.3389/fonc.2019.01187

**Published:** 2019-11-14

**Authors:** Wendong Yang, Zhongwei Li, Rong Qin, Xiaorui Wang, Huihui An, Yule Wang, Yan Zhu, Yantao Liu, Shijiao Cai, Shuang Chen, Tao Sun, Jing Meng, Cheng Yang

**Affiliations:** ^1^State Key Laboratory of Medicinal Chemical Biology and College of Pharmacy, Nankai University, Tianjin, China; ^2^Tianjin Key Laboratory of Early Druggability Evaluation of Innovative Drugs and Tianjin Key Laboratory of Molecular Drug Research, Tianjin International Joint Academy of Biomedicine, Tianjin, China; ^3^College of Life Sciences, Nankai University, Tianjin, China; ^4^Tianjin State Key Laboratory of Modern Chinese Medicine, Tianjin University of Traditional Chinese Medicine, Tianjin, China; ^5^Research and Development Center of TCM, Tianjin International Joint Academy of Biotechnology and Medicine, Tianjin, China

**Keywords:** YY1, angiogenesis, vascular endothelial growth factor A, transcription activation, hepatocellular carcinoma

## Abstract

Hepatocellular carcinoma (HCC) is a typical hypervascular solid tumor that requires neoangiogenesis for growth. The vascular endothelial growth factor (VEGF) is the most potent proangiogenic factor in neovascularization. The multifunctional Yin-Yang 1 (YY1) is involved in the regulation of tumor malignancy of HCC. However, the relationship between YY1 and endothelial cell-dependent tumor angiogenesis in HCC remains unclear. In this study, we observed that YY1 is positively correlated with microvessel density (MVD) and poor prognosis in HCC tissues. We further found that YY1 promotes the transcriptional activity of VEGFA by binding its promoter in HCC. The secreted VEGFA from HCC cells activates phosphorylation of VEGFR2 to promotes tube formation, cell migration, and invasion of vascular endothelial cells *in vitro*, and promotes tumor growth and angiogenesis *in vivo*. In addition, upregulation of YY1 enhanced resistance of bevacizumab in HCC cells. These results indicate that YY1 plays essential roles in HCC angiogenesis and resistance of bevacizumab by inducing VEGFA transcription and that YY1 may represent a potential molecular target for antiangiogenic therapy during HCC progression.

## Introduction

Hepatocellular carcinoma (HCC) is the fifth most frequent cancer in the world and the fourth leading cause of cancer-related death (1). HCC is the most common primary malignant liver tumor with abundant tumor vascular network, which provides the evidence for the clinical therapies targeted vascular endothelial growth factor (VEGF) for the treatment of unresectable HCC (2). Angiogenesis is critical to multiple tumor invasion and metastasis (3, 4). Targeted angiogenesis therapy is an important anti-tumor strategy at present and it is particularly important to understand the transcriptional regulation of tumor angiogenesis (5, 6). Angiogenesis involves complex signaling pathways (7–9). VEGFA is an important angiogenic factor secreted by both cancer cells and stromal infiltrating cells (10, 11). It is involved in the regulation of metastasis of many solid tumors and their neovasculature (12). VEGFA binds to two tyrosine kinase receptors of endothelial cells: VEGF receptor-1 (VEGFR1/Flt-1) and VEGFR2 (KDR). The function of VEGFR1 remains poorly defined, and VEGFR2 mediates proliferation and survival of endothelial cell (13–15). VEGFR2 is the major mediator of the mitogenic, angiogenic and permeability enhancing effects of VEGF (16). The high affinity between VEGFA and VEGFRs induces the proliferation, migration, and differentiation of vascular endothelial cells. Activated endothelial cells degrade the extracellular matrix, subsequently forming tubular structures and recruiting supporting cells to form stable vessels (17, 18).

Yin-Yang 1 (YY1) is a key transcription factor involved in cancer progression (19). YY1 is well-known for its dual roles in regulating gene expression, either as an activator or repressor, depending on the chromatin remodeling complexes it is recruited to (20, 21). Extensive evidence indicates that YY1 is an oncogene in various cancers, such as colorectal, prostate and breast cancer (22–24). There is reported that CXCR4/YY1 inhibition impairs VEGF network and angiogenesis during osteosarcoma malignancy (25). Competitive binding between Seryl-tRNA synthetase/YY1 complex and NFKB1 at the distal segment results in differential regulation of VEGF promoter activity during angiogenesis (26). In embryonic development, YY1 is responsible for maintaining VEGF in the developing visceral endoderm and that a VEGF-responsive paracrine signal, originating in the yolk sac mesoderm, is required to promote normal visceral endoderm development (27). Our previous studies showed that transcription complexes of YY1 promote malignant progression of hepatocellular carcinoma, and patients with high YY1 expression have poor prognosis (28). Although YY1 is involved in the regulation of tumor malignancy, its role and mechanism in tumor angiogenesis are rarely mentioned.

In our study, we analyzed the correlation between YY1 expression and MVD in HCC tissues and functional role of YY1 in HCC angiogenesis, and examined the underlying mechanism of YY1 regulated angiogenesis and drug sensitivity. This study may provide insights into a new potential therapeutic strategy and antitumor targets for HCC.

## Materials and Methods

### Cell Culture and Transfection

Human umbilical vein endothelial cells (HUVECs), human aortic endothelial cells (HAECs) and HepG2 cells were obtained from the Cell Bank of Shanghai Institute (Shanghai, China), Sciencell Research Laboratories (San Diego, USA) and KeyGen Biotech (Nanjing, China). HepG2 cells were cultured in RPMI1640 medium containing 10% fetal bovine serum (FBS) and 1% penicillin–streptomycin solution. After reaching 60–80% confluence, then the fresh medium was replenished. The supernatant was collected and centrifuged after 48 h incubation and stored at −80°C. HUVECs or HAECs were cultured in M-199 medium supplemented with endothelial cell growth supplement, 10% FBS, and 1% penicillin–streptomycin. After reaching 60–80% confluence, the HUVECs or HAECs were stimulated with condition medium (50% HepG2 with different treatment supernatants and 50% M-199) for 48 h and used as induced HUVECs, whereas normal HUVECs were used as control. Cells were maintained at 37°C in a humidified atmosphere with 5% CO_2_. All the plasmids were transfected into cells by Lipofectamine™ 2000 (Invitrogen, 11668019) in accordance with the manufacturer's instructions. Each experiment was performed in triplicate and repeated at least three times.

### Luciferase Activity Assays

HUVECs were seeded in 96-well plates. After 24 h, a pGL3 promoter vector containing the VEGFA promoter region was co-transfected with the indicated plasmids. The luciferase activities were detected using a dual-luciferase reporter gene assay kit (RG027, Beyotime) after 48 h transfection. Renilla luciferase activity was used as an internal standard. Each experiment was conducted in triplicate.

### Cell Invasion Assays

Matrigel (Corning, 354234) was diluted (1:2) in serum-free media and seeded in a 24-well transwell chamber (JET, TCS013024). After HUVECs or HAECs were incubated with the indicated cell supernatants for 48 h, ~1 × 10^5^ HUVECs or HAECs were seeded on the matrigel, and FBS was added to a 24-well plate located below the chamber to serve as a chemoattractant. After 24 h, invasive cells were stained with 0.1% crystal violet for 10 min. Images were obtained using a phase contrast microscope.

### Wound Healing Assay

HUVECs or HAECs stimulated with condition medium (50% HepG2 with different treatment supernatants and 50% M-199) were seeded in wells for 12 h at 37°C. A micropipette tip was used to scrape a straight line in each well. After 24 and 48 h, the migration of cells was analyzed by comparing the wound distance ratio at 0 h. Each experiment was performed in triplicate.

### Tube Formation Assay

HUVECs or HAECs suspended in conditioned medium were seeded onto a 48-well plate coated with Matrigel (Corning, 354234) and incubated for 8 h at 37°C. Tube formation was observed at 3 h post-treatment. The number of tubes for each treatment was quantified. This experiment was independently repeated thrice and four random fields were observed every time.

### Western Blot (WB) Analysis

Cells were washed with phosphate-buffered saline (PBS) and lysed in ice-cold lysis buffer containing protease inhibitor cocktail (Sigma) for 30 min. Lysates were separated by SDS-PAGE and transferred onto a 0.45 μm PVDF membrane. After transferring, the membranes were blocked with 5% BSA at room temperature with shaking for 2 h. Membranes were incubated with anti-YY1 (1:1,000, Santa, sc-7341), anti-VEGFA (1:1,000, Affinity, DF7470), anti-pVEGFR2 (1:1,000, Affinity, AF3281), and anti-GAPDH (1:4,000, Affinity, T0004) diluted with 5% BSA overnight at 4°C. Then, the membranes were washed three times with TBST for 10 min at room temperature and incubated with secondary antibody at room temperature for 2 h. Protein expression was assessed with enhanced chemiluminescent substrate (Millipore, USA) and by exposure to chemiluminescent film.

### Immunofluorescence

HUVECs or HAECs incubated with the indicated supernatants were grown on glass slides until 70–80% confluent. The cells were washed three times with 1 × PBS. They were fixed in 4% PFA at room temperature for 20 min. Subsequently, the cells underwent blocking and permeabilization with 5% BSA containing 0.1% Triton X-100 for 30 min at room temperature. They were incubated overnight at 4°C with pVEGFR2 antibody (1:200, Affinity, AF3281) and then incubated with TRITC- labeled secondary antibodies (1:50, KeyGEN BioTECH) for 1 h at room temperature. Each step was followed by two 5-min washes in PBS. The prepared specimens were counterstained with DAPI (Solarbio, S2110) for 30 min. Images were acquired using a Leica confocal microscope.

### qRT-PCR

Total RNAs were extracted from different treatment cells using TRIzol reagent (Invitrogen, 15596026). FastQuant RT kit (TIANGEN, R6906) was utilized to obtain cDNA following the manufacturer's protocol. An SYBR Green Kit (TIANGEN, FP205) was used for transcript quantification with specific primers on QuantStudio™ 6 (Life Technologies, Singapore). The samples were run in triplicate in each experiment, and a housekeeping gene (GAPDH) was used as an internal standard. The 2^−ΔΔCT^ method was applied to quantify the relative gene expression. The sequences of gene-specific primers were as follows: VEGFA: F:5′-GCCTTGCCTTGCTGCTCTAC-3′; R:5′-TGATTCTGCCCTCCTCCTT CTG-3′; GAPDH: F:5′-GTCCACTGGCGTCTTCAC-3′; R:5′-CTTGAGGCTGTTGTC ATACTTC-3′. GAPDH served as loading control.

### Enzyme-Linked Immunosorbent Assay (ELISA)

To detect VEGFA in culture supernatants, ELISA was carried out with ELISA kits (Beyotime, PV963) in accordance with the manufacturer's recommendations.

### Three-Dimensional Minitumor Generation

HepG2 cells with different treatment cocultured with HUVECs or HAECs at a 2:1 mix ratio. To characterize the tumor-like spheroids formed by HepG2 and HUVECs, the cells were stained with DIO (Beyotime, C1038) and DIL (BestBio, BB-441921), respectively, following the manufacturers' instructions. HepG2 and HUVECs were spun down, resuspended, and then divided by 150 μL into wells of a U-shaped 96-well suspension plate (Greiner Bio-One, Stonehouse, UK). The plate was incubated at 37°C for 48 h to allow for spheroid formation (29). A laser scanning confocal microscope (ZEISS) was used to examine the structural organization of tumor spheroids. The integrated intensity of tumor spheroids was analyzed by ImageJ.

### ChIP-seq Assay and Analysis

Approximately 1 × 10^7^ HepG2 cells were freshly harvested and fixed in 1% formaldehyde/medium buffer for 10 min at room temperature. Fixation was stopped by the addition of glycine to a final concentration of 250 mM. Cell pellets were resuspended in cell lysis buffer containing 1 × Protease Inhibitor Cocktail II and then incubated for 15 min on ice. They were then pipetted for dissociation and pelleted by centrifugation at 800 g at 4°C for 5 min. Approximately 1 mL of nuclear lysis buffer was added to resuspend the cell pellets. To ensure sonication, bioanalyzer analysis was performed. The chromatin fraction was incubated with the indicated antibody overnight at 4°C. The protein/DNA complexes were reverse cross-linked to obtain free DNA. Spin columns were utilized to purify DNA and were then quantified by qPCR. The samples were sequenced by Genergy Biotechnology. Chromatin immunoprecipitation sequencing (ChIP-seq) data were obtained from Cistrome Data Browser (http://cistrome.org/db). IGV software was used to analyze ChIP-seq data and obtain ChIP peak. The primer pair was tested for spanning regions in the VEGFA promoter: F: 5′-CACTGACTAACCCCGGAACC-3′; R: 5′-GGAGTGACTGGGGTCCTTT G-3′.

### Xenograft Tumor Model

BALB/c nude mice (weighing ~20 g, 4–6 weeks) were randomly divided into Ctrl, YY1, siYY1, and YY1 + Beva groups (*n* = 3 male + 3 female per group). The mice were injected with 1 × 10^6^ HepG2 cells or stably overexpressed YY1 subcutaneously in the mid-dorsal region. When the tumor size reached about 200 mm^3^, the siYY1 group was intratumorally injected with siYY1 loaded in nanoparticles. The YY1 + Beva group was treated with 2 mg/kg bevacizumab twice per week by intraperitoneal injection for 24 days. Solvent buffer at the same volume was used in other groups. The tumor sizes were measured and calculated according to a standard formula every 3 days.

This study was carried out in accordance with the principles of the Basel Declaration and recommendations of International Association of Veterinary Editors guidelines, Nankai University Ethics Committee. The protocol was approved by the Nankai University Ethics Committee.

### Immunohistochemistry (IHC) Assay

Paraffin sections of human HCC samples and tumor tissues were deparaffinized with xylene and dehydrated with decreasing concentration of ethanol. The endogenous peroxidase activity was blocked with 3% hydrogen peroxide. Microwave antigen retrieval technique was used. Non-specific antigen sites were blocked using normal goat serum at room temperature for 20 min. Primary antibodies, including YY1 (1:100, Santa, sc-7341), pVEGFR2 (1:100, Affinity, AF3281), and CD31 (1:25, abcam, ab9498), were incubated in a humidified chamber overnight at 4°C. HRP-polymer anti-mouse or rabbit IHC kit (Maixin Biotech, China) was utilized to incubate secondary antibody. Samples were developed with diaminobenzidine reagent and counterstained with hematoxylin. The microvessel density (MVD) were quantified using ImageJ software on the basis of CD31 staining.

### Patient Samples

HCC tissue contains 26 cases were collected Tianjin Medical General Hospital and Tumor Hospital of Tianjin within 5 years. The donor was completely informed and each specimen from patients were obtained with hospital and the individual consent. All tissues were harvested under the highest ethical standards.

This study was carried out in accordance with the recommendations of Ethical Review Measures for Biomedical Research Involving Human Beings (Trial Implementation), Nankai University Ethics Committee. The protocol was approved by the Nankai University Ethics Committee. All subjects gave written informed consent in accordance with the Declaration of Helsinki.

### Statistical Analysis

GraphPad Prism 7.0 software (GraphPad Software, Inc., San Diego, CA, USA) and SPSS v19.0 (IBM, Armond, NY, USA) were utilized to perform statistical analyses. Two-tailed unpaired Student's *t*-test was used for comparing two groups of data. One-ANOVA was used to compare multiple groups of data. Pearson's correlation was used for relevance analysis. Kaplan–Meier analysis was used for survival analysis. Data from biological triplicate experiments were presented with error bar as mean ± SD. Statistical significance was considered at *P* < 0.05.

## Results

### YY1 Was Associated With Angiogenesis of HCC

**Figure 1:**
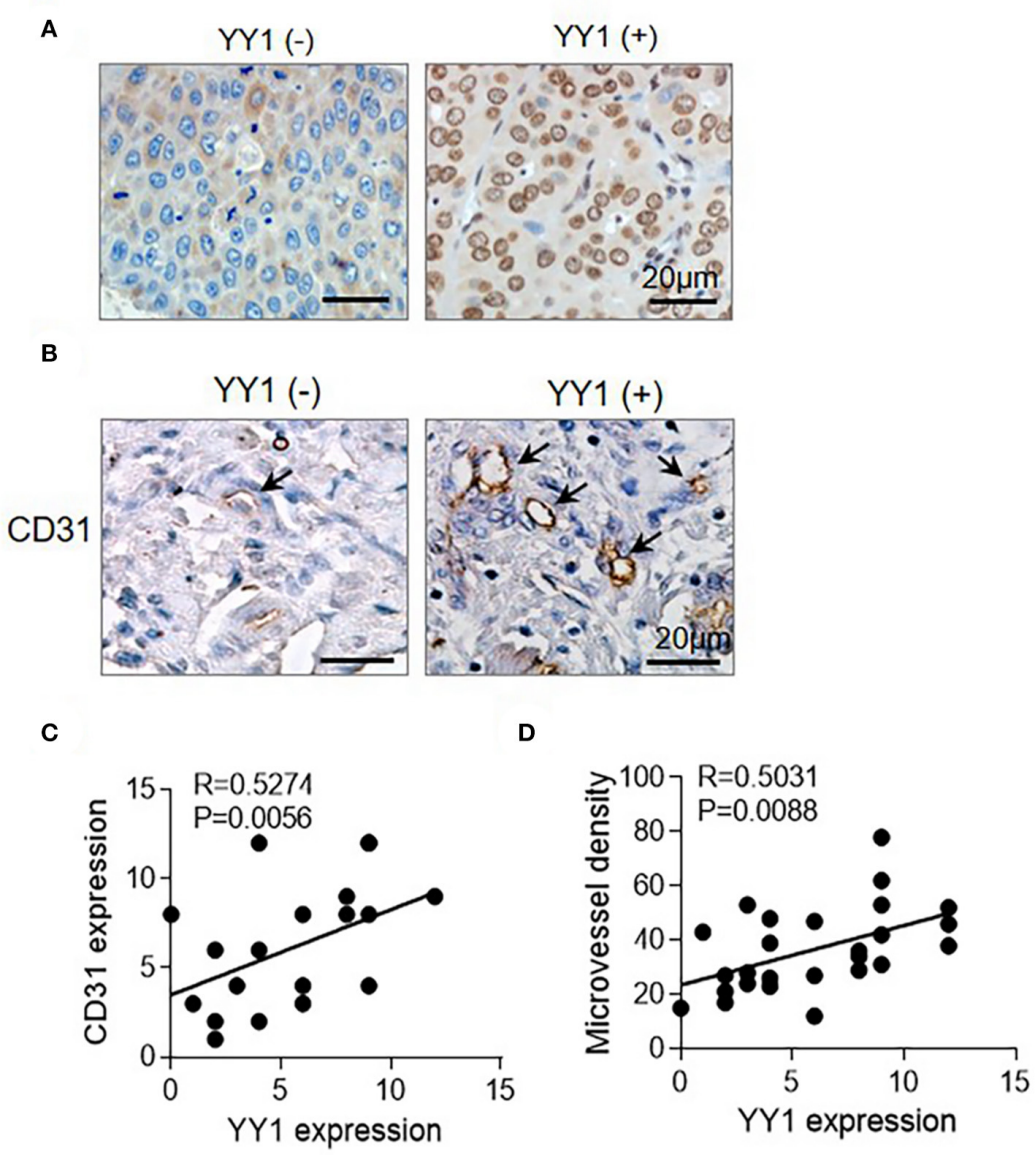
YY1 was associated with HCC angiogenesis. **(A)** Representative images of IHC staining for YY1 of human HCC tissues at different stages (left, stage I; right, stage IV). Scale bar = 20 μm. **(B)** MVD measured by immunostaining for CD31 in YY1-negative and positive HCC tissues. Black arrows indicate microvessels. Scale bar = 20 μm. **(C)** CD31 and YY1 stains were quantified and the correlation was analyzed (correlation coefficient: *R* = 0.5274, *P* = 0.0056). **(D)** MVD and YY1 stains were quantified and the correlation was analyzed (correlation coefficient: *R* = 0.5031, *P* = 0.0088).

In our previous research, we confirmed that YY1 correlates closely to HCC metastasis and recurrence (28). We analyzed 26 HCC cases by IHC analysis, angiogenesis was showed CD31 staining positive. The expression level of YY1 and angiogenesis were higher in high-degree of malignancy tissues than in low-degree of malignancy (Figures 1A,B). Pearson's correlation and linear regression analysis showed that the expression levels of YY1 and CD31 were positively correlated (Figure 1C). The MVD was calculated by IHC staining with anti-CD31. The result showed that YY1 was positively correlated with MVD in HCC (Figure 1D).

### YY1 Indicated Tumor Malignancy in HCC

**Figure 2:**
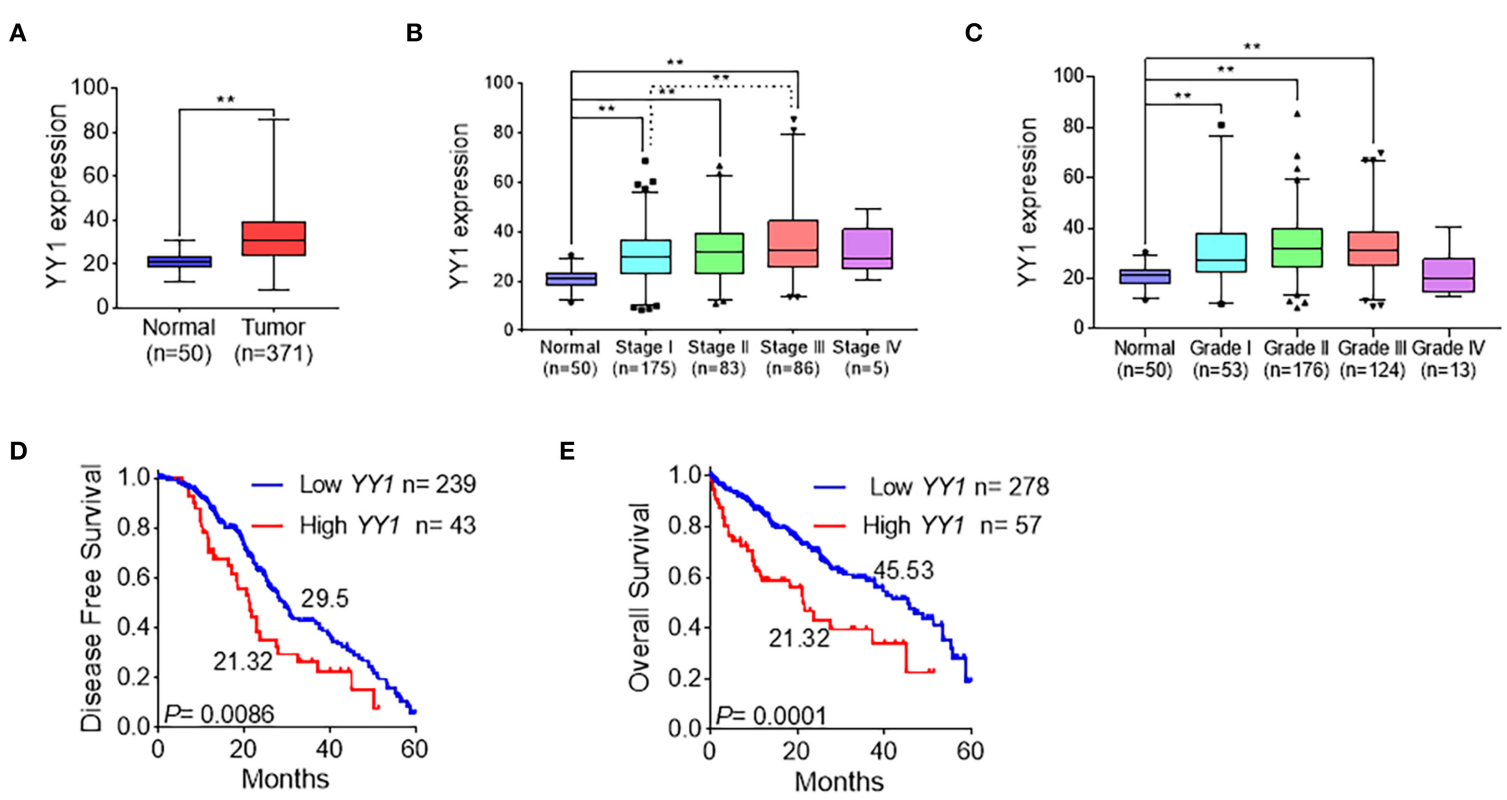
YY1 indicated tumor malignancy in HCC. **(A)** Expression level of YY1 in primary tumors (*n* = 371) and normal liver tissues (*n* = 50) on the basis of the TCGA dataset. **(B)** Analysis of the expression levels of YY1 in TCGA LIHC samples on the basis of clinical stages. **(C)** Analysis of the expression levels of YY1 in TCGA LIHC samples on the basis of pathology grade. **(D)** Kaplan-Meier curve shows the 5-year disease-free survival rate of TCGA LIHC samples classified by YY1 expression. **(E)** High YY1 expression was significantly associated with poor overall survival in TCGA LIHC samples. ^**^P < 0.01.

To explore the clinicopathologically relevant feature of YY1, the LIHC dataset from TCGA was analyzed. The expression level of YY1 in HCC was higher than that in normal tissues (Figure 2A), which suggests that YY1 may promote the malignant progression of HCC. Further analysis of these data showed that YY1 expression was positively correlated with clinical stages and pathological grades, except stage IV, which may due to few patients in the IV groups (Figures 2B,C). Meanwhile, disease-free survival and overall survival analysis demonstrated that the high expression of YY1 in HCC indicates a poor clinical prognosis (Figures 2D,E). These results suggested that YY1 promotes the malignant progression of HCC.

### YY1 Binds to VEGFA Promoter to Enhance VEGFA Expression in HCC Cells

**Figure 3:**
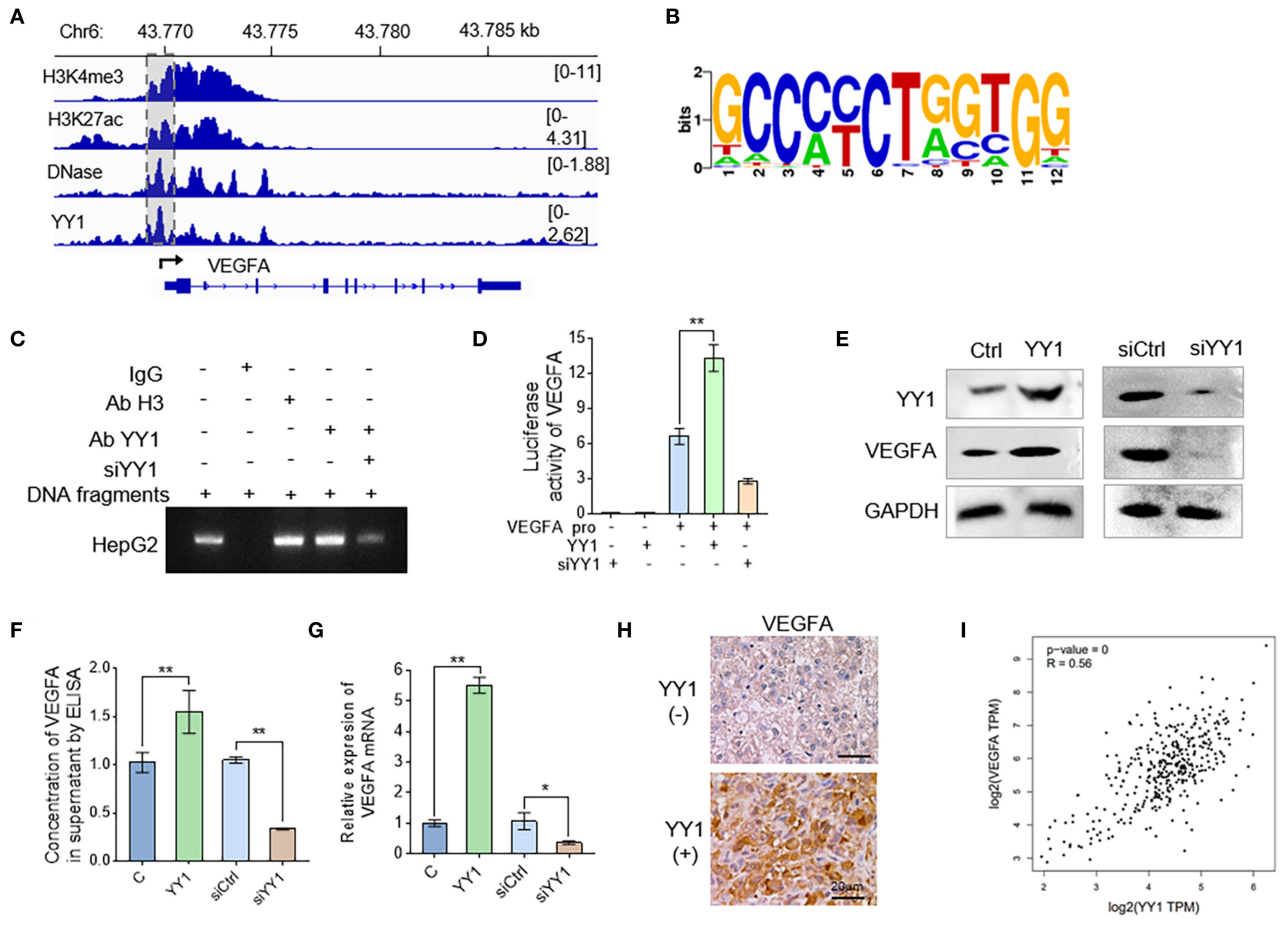
YY1 binds to VEGFA promoter to enhance VEGFA expression in HCC cells. **(A)** Genomic tracks for ChIP-seq around VEGFA and location of promoter (pink area). **(B)** Analysis of motifs enriched in YY1 ChIP-seq. **(C)** HepG2 cells were treated with YY1 overexpression vectors and YY1siRNA. Cellular extracts were prepared for ChIP assays with anti-YY1. **(D)** HepG2 cells were transiently transfected with VEGFA-dependent reporter gene plasmids. Luciferase activity was measured when cells were overexpressed with or knocked down of YY1. **(E)** WB analysis showed the VEGFA expression levels in HepG2 cells overexpressed with or knocked down of YY1. **(F)** ELISAs were used to determine the VEGFA concentrations in the supernatants of the HepG2 cells transfected with YY1 and siYY1. **(G)** The mRNA levels of VEGFA in HepG2 cells transfected with YY1 or siYY1were measured by qRT-PCR. **(H)** VEGFA expression levels in YY1-negative and YY1-positive HCC tissues. (scale bar = 20 μm). **(I)** Correlation analysis between YY1 and VEGFA in TCGA database (*R* = 0.56, *P* = 0). ^*^P < 0.05, ^**^P < 0.01.

VEGFA is a prominent factor involved in the acquisition of endothelial cell-dependent angiogenesis. In order to elucidate the underlying mechanism that YY1 induces angiogenesis, we detected the effects of YY1 on VEGFA expression. To explore the regulation of YY1 to VEGFA, we analyzed the H3K4me3, H3K27ac, DNase, and YY1 ChIP-seq data of Cistrome Data Browser database. The results showed that YY1 binds to VEGFA promoter (Figure 3A). ChIP-seq was used to further analyze the DNA-binding motif of YY1 on the VEGFA promoter (Figure 3B). ChIP-PCR analysis was carried out on HepG2 cells by using specific antibodies against YY1, showing the occupancy of YY1 on the VEGFA promoter, which validated the ChIP-seq results (Figure 3C and Figure S1). In addition, the effect of YY1 on the promoter activities of VEGFA were detected by dual-luciferase reporter system. YY1 increased VEGFA promoter activities, whereas YY1 knockdown decreased VEGFA promoter activities (Figure 3D). The protein expression in cells (Figure 3E and Figure S2) and secreted VEGFA (Figure 3F) were consistent with the mRNA expression (Figure 3G), and the results showed that VEGFRA expression levels increased after YY1 overexpression and decreased after YY1 silence. In addition, we confirmed the correction between YY1 and VEGFA in HCC tissues. IHC staining showed that high YY1 expression levels exhibited extremely strong stain of VEGFA in HCC tissues (Figure 3H). Correlation analysis showed that YY1 was associated with VEGFA expression in HCC tissues of TAGA database (*R* = 0.56, *P* = 0) (http://gepia.cancer-pku.cn) (Figure 3I). In summary, YY1 binds VEGFA promoter to upregulate its transcription activities, protein expression, and secretion in HCC.

### YY1 Stimulated HCC Cell Culture Media Accelerated Endothelial Cells Neovascularization

**Figure 4:**
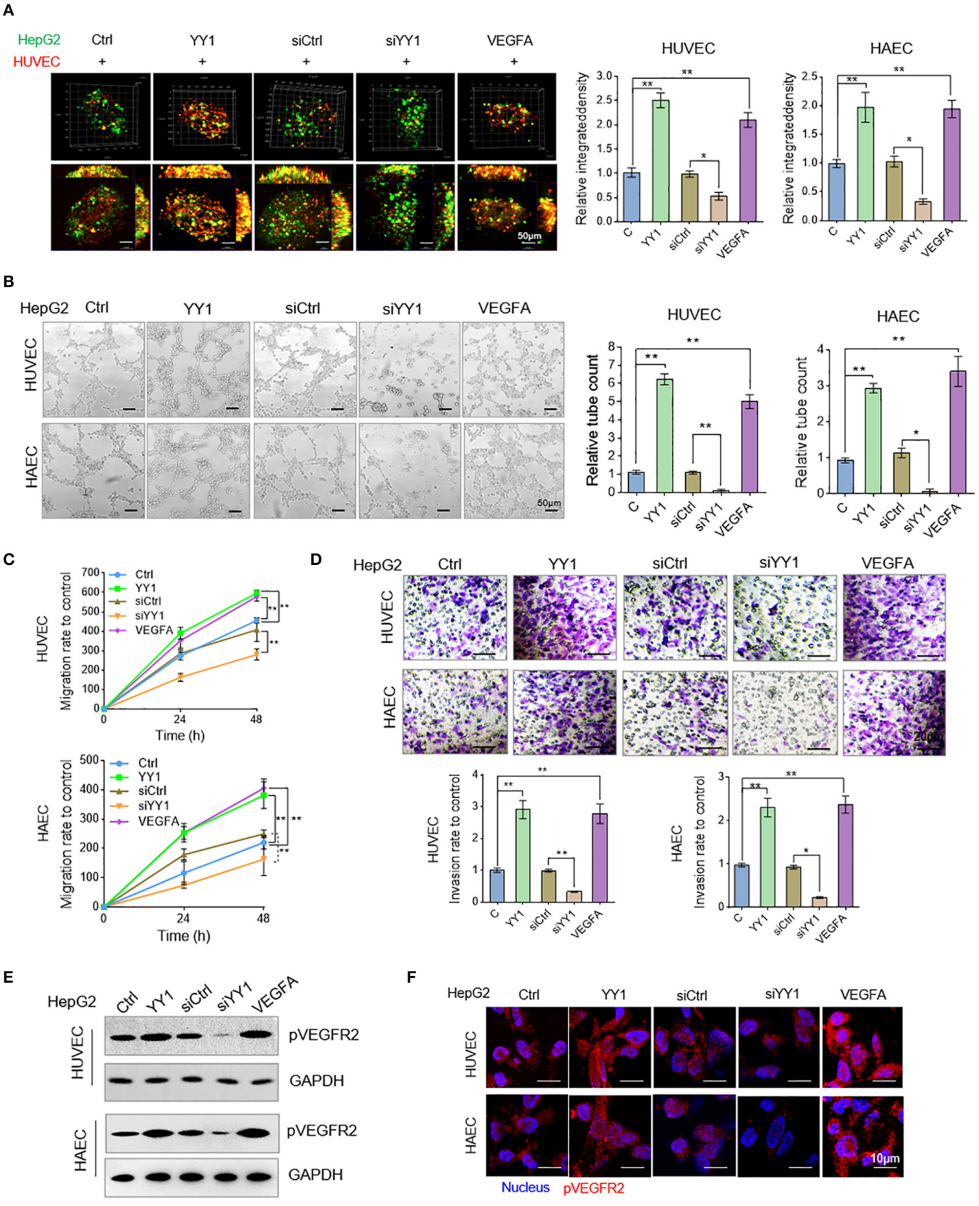
YY1 stimulated HCC cell culture media accelerated endothelial cells neovascularization. **(A)** HUVECs and HAECs (red) and HepG2 cells (green) co-cultured in a 1:2 ratio and formed three-dimensional spheroids. Images were taken with a laser scanning confocal microscope, scale bar = 50 μm. **(B)** Representative image (left) of the formation of HUVECs and HAECs tubes following an incubation with supernatants collected from the indicated cells. Tube formation quantification were analyzed (right). Scale bar = 50 μm. **(C)** HUVECs and HAECs migration were detected after an incubation with supernatants collected from the indicated cells. **(D)** HUVECs and HAECs invasion were detected following an incubation with supernatants collected from the indicated cells. Scale bar = 20 μm. **(E)** WB analyzed pVEGFR2 expression in HUVECs and HAECs treated with conditioned media. **(F)** Immunofluorescence of pVEGFR2 expression in HUVECs and HAECs treated with conditioned media. Scale bar = 10 μm. ^*^P < 0.05, ^**^P < 0.01.

Angiogenesis was measured by *in vitro* tube formation assay. To examine the effects of YY1 in HCC cells on HUVEC or HAECs tube formation, we detected the morphologies of vessel-like structure of co-cultured GFP-labeled HepG2 cells and RFP-labeled HUVECs or HAECs in three-dimensional culture. The showed that YY1 and VEGFA could significantly induce the formation of vessel-like structures more than that in normal condition and siYY1 could reduce vessel-like structure compared with siCtrl (Figure 4A). Then, the tube formation, migration and invasion were detected in HUVECs or HAECs that cultured with condition medium from supernatants of HepG2 cells transfected with YY1 and YY1 siRNA or treated with VEGFA for 48 h. The results showed that the conditioned medium from YY1-overexpression treatment significantly enhanced HUVEC or HAECs tube formation and knockdown YY1 downregulated tube formation (Figure 4B). Conditioned medium from YY1 overexpression or VEGFA treatment promoted HUVEC or HAECs migration and invasion. However, YY1 knockdown inhibited the migration and invasion of HUVECs (Figures 4C,D and Figure S3). Phosphorylation level of VEGFR2 in HUVECs or HAECs were increased after conditioned medium from HepG2 cell with YY1 overexpression or VEGFA treatment. Conversely, phosphorylation level of VEGFR2 decreased after YY1 knockdown (Figure 4E and Figure S4). This result was validated by immunofluorescence staining (Figure 4F).

### Bevacizumab Blocked the Promotive Effect of YY1 on Tube Formation Through VEGFA

**Figure 5:**
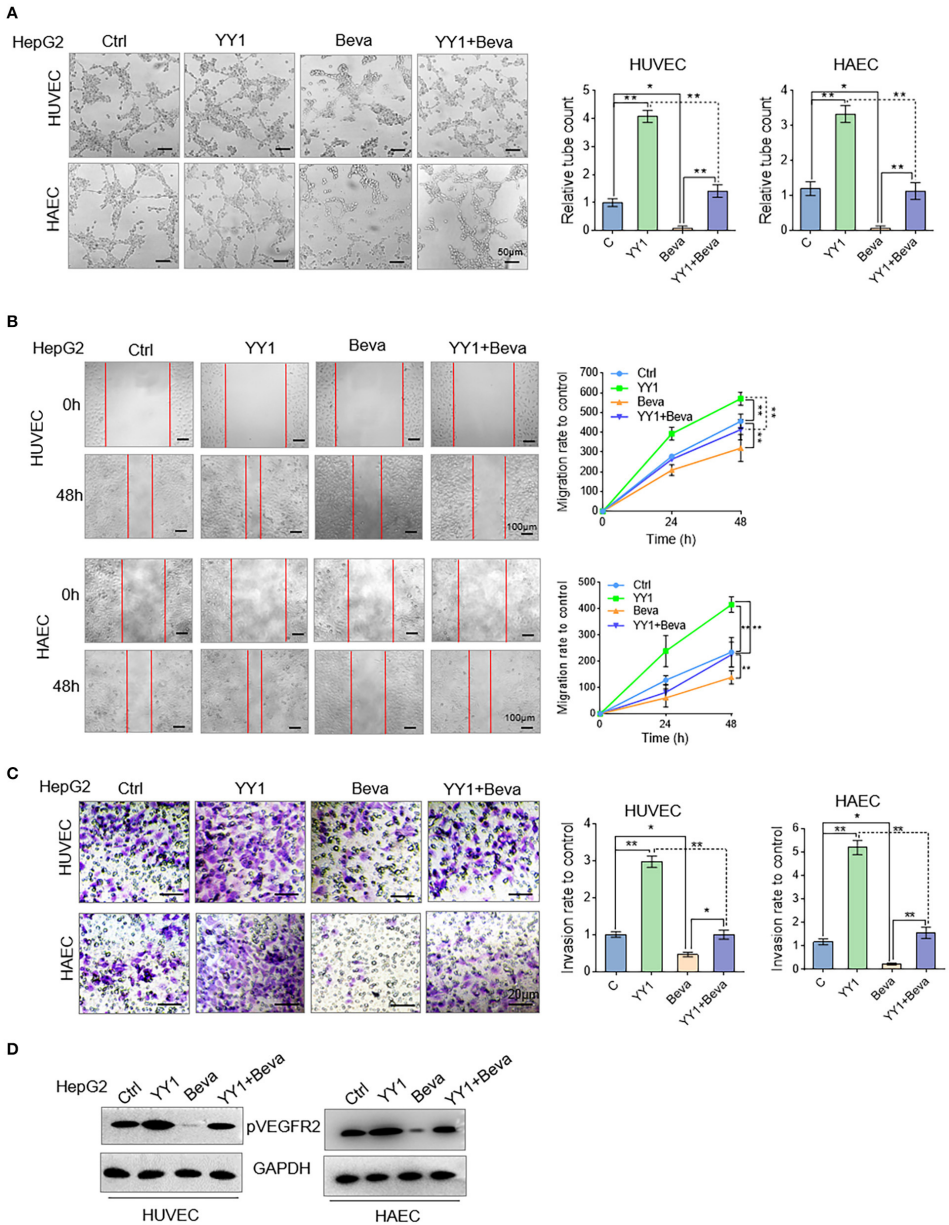
Bevacizumab blocked the promotive effect of YY1 on tube formation through VEGFA. **(A)** Tube formation of HUVECs and HAECs cultured in the indicated cells treated with or without bevacizumab or transfected with YY1. Scale bar = 50 μm. **(B,C)** Migration and invasion of HUVECs and HAECs cultured in the indicated cells treated with or without bevacizumab or transfected with YY1. **(D)** WB analysis showed pVEGFR2 expression levels in HUVECs and HAECs treated with conditioned media. ^*^P < 0.05, ^**^P < 0.01.

Bevacizumab is an anti-VEGFA monoclonal antibody (30). In HCC cells, the effects of YY1 upregulation on bevacizumab resistance through the VEGFA transcriptional activation were detected. YY1-overexpression or control HCC cells were treated with 250 μg/mL bevacizumab for 48 h and supernatant of culture medium were collected. Tube formation assays were performed by treating the HUVECs or HAECs with the indicated cell supernatants. Ectopic expression of YY1 significantly increased the tube formation by HUVECs or HAECs. Bevacizumab blocked the promotive effect of conditioned medium with YY1-overexpressing on tube formation (Figure 5A). In addition, we treated migration and invasion in HUVECs or HAECs treated with the same conditioned medium and the results showed that bevacizumab blocked the promotive effect of conditioned medium with YY1-overexpressing on migration and invasion (Figures 5B,C). WB analysis confirmed that bevacizumab inhibited the phosphorylation level of VEGFR2, and bevacizumab also blocked the effect of YY1 on the phosphorylation of VEGFR2 (Figure 5D and Figure S5). These results showed that bevacizumab blocked the promotive effect of YY1 on tube formation and YY1 overexpression increased bevacizumab resistance by inducing VEGFA transcription.

### YY1 Enhanced Tumor Vascularization in HCC Xenograft Model by Promoting VEGFA Expression

**Figure 6:**
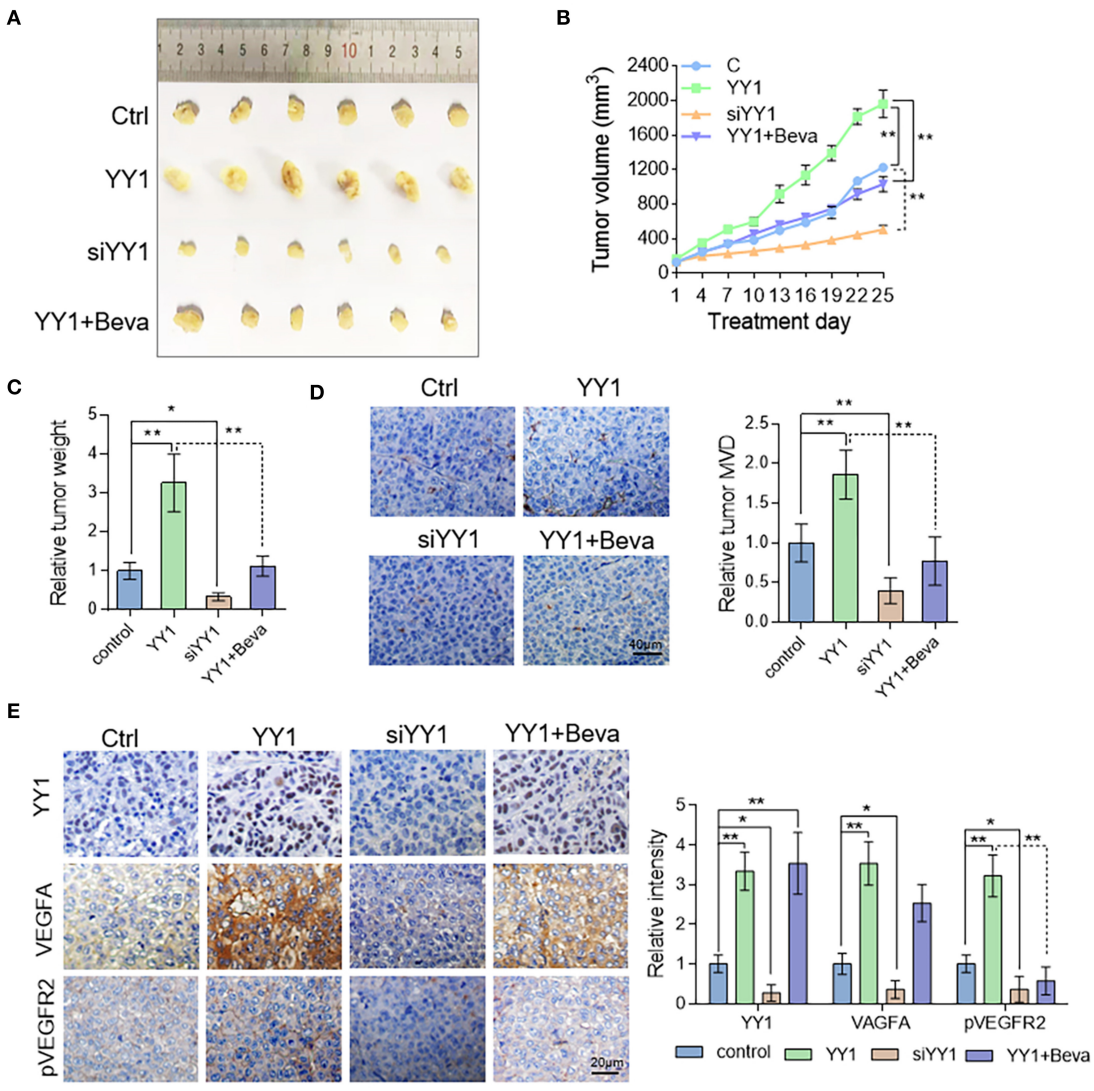
YY1 enhanced tumor vascularization in HCC xenograft model by promoting VEGFA expression. **(A)** Images of subcutaneous tumors of Ctrl, YY1, siYY1, and YY1 + bevacizumab group mice (*n* = 6/per group). **(B)** Tumor size was measured starting from bevacizumab treatment. **(C)** Tumor weight in control, YY1 and siYY1 and YY1 + bevacizumab groups. **(D)** Analysis of MVD on the basis of CD31 staining of tumor tissue. Scale bar = 40 μm. **(E)** Immunohistochemical staining of YY1, VEGFA, and pVEGFR2 expression levels in tumor tissue of the Ctrl, YY1, siYY1, and YY1 + bevacizumab groups. Scale bar = 20 μm. ^*^P < 0.05, ^**^P < 0.01.

To assess the effect of YY1 on tumor angiogenesis *in vivo*, nude mice were subcutaneously implanted HepG2 cells. Tumor-bearing nude mice in bevacizumab group were treated with 2 mg/kg bevacizumab twice per week by intraperitoneal injection. Compared with the control, YY1 overexpression promoted tumor growth (Figures 6A,B), tumor weight (Figure 6C) and MVD, which indicated by CD31-positive cells (Figure 6D). The opposite results were obtained after silencing YY1 expression. Bevacizumab treatment (2 mg/kg) abrogated the promotive effect of YY1 on the tumor volume and *in vivo* angiogenesis. Next, the protein expression of YY1, VEGFA, and pVEGFR2 in xenograft tumors were analyzed by immunohistochemistry. As shown in Figure 6E, the expression levels of VEGFA and phosphorylation level of VEGFR2 were increased in YY1 overexpressed group and decreased in YY1 silenced group that that in control group. Bevacizumab blocked the upregulated effect of YY1 on the phosphorylation of VEGFR2 *in vivo*. These results suggested that YY1 contributed to endothelial cell-dependent angiogenesis *in vivo* through promote VEGFA expression.

## Discussion

Angiogenesis is associated with tumor metastasis, malignancy, and poor clinical prognosis of patients (31). Considering the association of aggressive tumors and angiogenesis, developing targeted therapies according angiogenesis formation and induction mechanism is important.

YY1 promotes epithelial–mesenchymal transition in HCC (28); however, the relationship between YY1 and endothelium-dependent angiogenesis has rarely reported. Analysis of the clinical stage and pathological grade in LIHC cases of TCGA database showed that YY1 expression is a risk factor that determines the survival of HCC patients. Kaplan–Meier analysis revealed that the disease-free survival and overall survival time in YY1-positive HCC patients were shorter than those in YY1-negative patients. The results showed that YY1 expressed highly in tumor tissues than normal tissues and upregulated in HCCs with a high degree of malignancy. YY1 plays an important role in poor prognosis. The results of CD31-positive endothelial cell-dependent microvessel density showed that YY1 expression was positively correlated with MVD.

**Figure 7:**
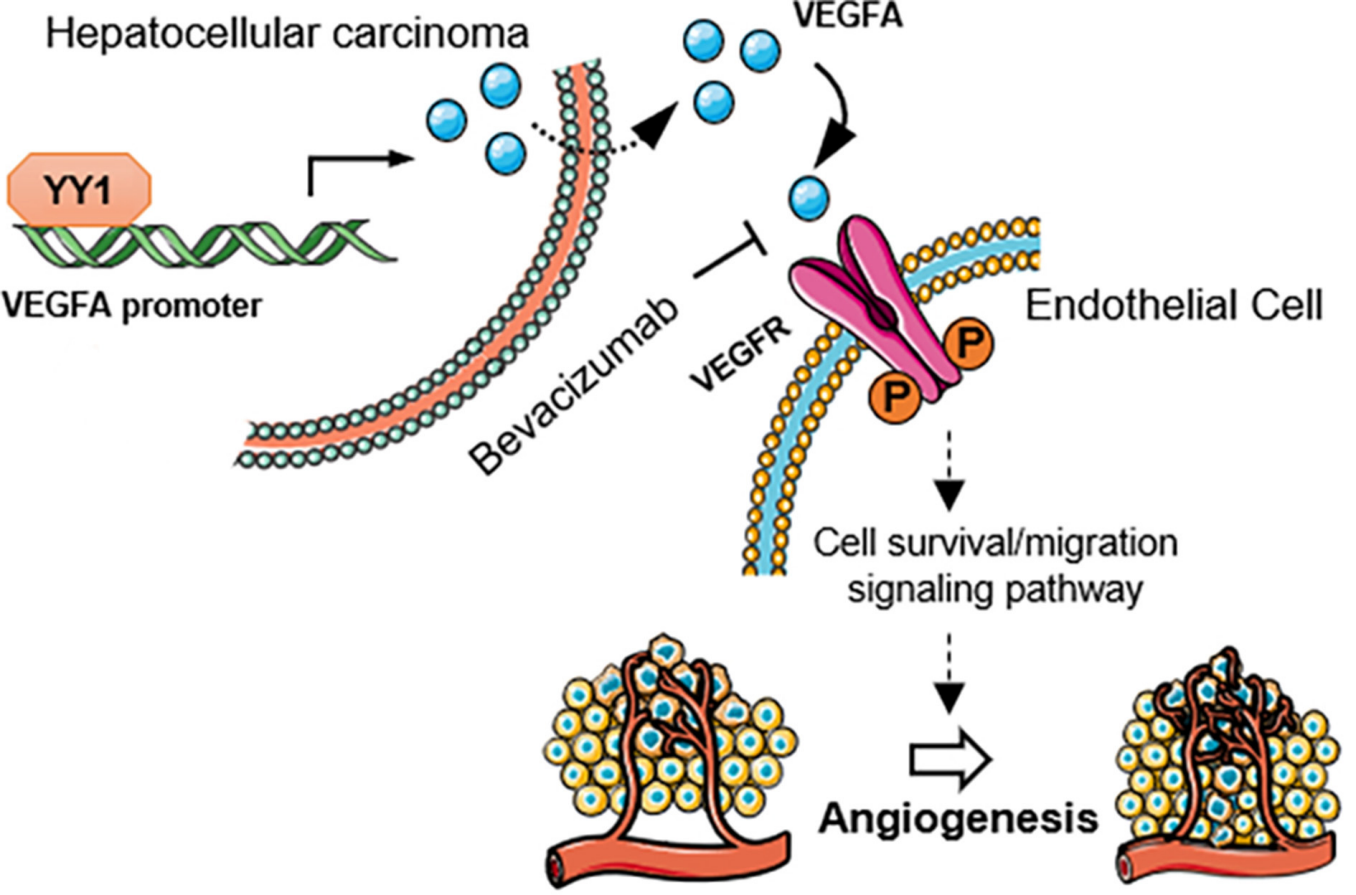
Schematic diagram of the mechanism that YY1 promotes angiogenesis in HCC by activating VEGFA transcription. Secretion of VEGFA stimulated by YY1 promoted phosphorylation level of VEGFR2 in endothelial cells, which activated VEGFR2 associated angiogenesis signaling pathway.

The growth and maintenance of angiogenesis were modulated by various growth factor pathways (32). VEGFA is one of most critical growth factors that regulates angiogenesis (33). We also detected the correlation of YY1 and VEGFA in HCC *in vitro*. YY1 was positively related to VEGFA, which are crucial to tumor angiogenesis, promote endothelial cell proliferation, and increase vascular permeability. YY1 may promote angiogenesis formation by promoting VEGFA expression in HCC. We further found that YY1 interacts with the promoter of VEGFA and enhances its transcriptional activity in HCC cells. YY1 overexpression increased VEGFA transcriptional activity, whereas YY1 knockdown decreased VEGFA expression and secretion. HUVECs co-cultured with conditioned HCC cells or cultured with conditioned medium from HCC cells, and secreted VEGFA from HCC cells promoted tube formation, migration and invasion of HUVECs *in vitro*. These results showed that the exogenous overexpression of YY1 in HCC cells increased the secretion of VEGFA and continued to activate the VEGFR signaling pathway in endothelial cells. After VEGFA treatment, more receptors were induced to combine with VEGFA, leading to the activation of the VEGFA/VEGFR pathways. Secretion of VEGFA stimulated by YY1 promoted phosphorylation level of VEGFR2 in HUVECs, which activated VEGFR2 associated angiogenesis signaling pathway (Figure 7).

VEGFA plays a critical role in angiogenesis, and its expression is upregulated in HCC cells. Blocked of VEGFA signaling inhibits tumor growth and angiogenesis (34). Bevacizumab, the first and most commonly used anti-angiogenic drug, prevents the activation of VEGFR signaling by specifically targeting VEGFA to Ferrara et al. (16), Kerr (35), and Ramezani et al. (36) Although bevacizumab is a molecular-targeted therapy and served as the first-line treatment option for metastatic colorectal cancer, breast cancer, renal cell carcinoma, and advanced non-small cell lung cancer (32), its resistance limits its therapeutic efficacy in the clinical treatment. Our results showed that bevacizumab blocked the promotive effect of YY1 on angiogenesis and YY1 overexpression increased bevacizumab resistance by inducing VEGFA transcription. *In vivo*, YY1 promoted tumor growth, and angiogenesis formation also relied on VEGFA. This finding indicates that YY1 promotes angiogenesis formation depending on the transcription activation of YY1 on VEGFA. Therefore, YY1 can be used as a potential target of angiogenesis.

In conclusion, our data indicated that YY1 promotes endothelial cell-dependent tumor angiogenesis by promoting VEGFA transcription of HCC *in vitro* and *in vivo*. This work also provides a potential antitumor therapy for inhibiting angiogenesis by targeting YY1 in HCC.

## Data Availability Statement

The datasets generated for this study will not be made publicly available. There is no omics dataset which requires submission to public databases. The datasets for this study were from TCGA public databases and Cistrome Data Browser database.

## Ethics Statement

The animal study was reviewed and approved by Laboratory Animal Ethics Committee of Nankai University.

## Author Contributions

TS and JM conceived and designed the projects. JM and ZL wrote the manuscript. WY, ZL, RQ, and HA performed the experiments. CY, TS, SCh, YW, YZ, YL, and SCa provided technical and material support. JM, ZL, and XW performed the data analysis.

### Conflict of Interest

The authors declare that the research was conducted in the absence of any commercial or financial relationships that could be construed as a potential conflict of interest.
