# Corrigendum: YY1 Promotes Endothelial Cell-Dependent Tumor Angiogenesis in Hepatocellular Carcinoma by Transcriptionally Activating VEGFA

**DOI:** 10.3389/fonc.2021.828861

**Published:** 2022-01-13

**Authors:** Wendong Yang, Zhongwei Li, Rong Qin, Xiaorui Wang, Huihui An, Yule Wang, Yan Zhu, Yantao Liu, Shijiao Cai, Shuang Chen, Tao Sun, Jing Meng, Cheng Yang

**Affiliations:** ^1^ State Key Laboratory of Medicinal Chemical Biology and College of Pharmacy, Nankai University, Tianjin, China; ^2^ Tianjin Key Laboratory of Early Druggability Evaluation of Innovative Drugs and Tianjin Key Laboratory of Molecular Drug Research, Tianjin International Joint Academy of Biomedicine, Tianjin, China; ^3^ College of Life Sciences, Nankai University, Tianjin, China; ^4^ Tianjin State Key Laboratory of Modern Chinese Medicine, Tianjin University of Traditional Chinese Medicine, Tianjin, China; ^5^ Research and Development Center of TCM, Tianjin International Joint Academy of Biotechnology and Medicine, Tianjin, China

**Keywords:** YY1, angiogenesis, vascular endothelial growth factor A, transcription activation, hepatocellular carcinoma

In the original article, there was a mistake in **Figure 4B** as published. The HAEC tubes formation following an incubation with supernatants collected from the YY1 overexpression cells was placed mistakenly using the same image of HUVECs tubes with same treatment in **Figure 4B**. The mistake was inadvertently introduced in the preparation of revision manuscript. The corrected **Figure 4** appears below.

**Figure 4:**
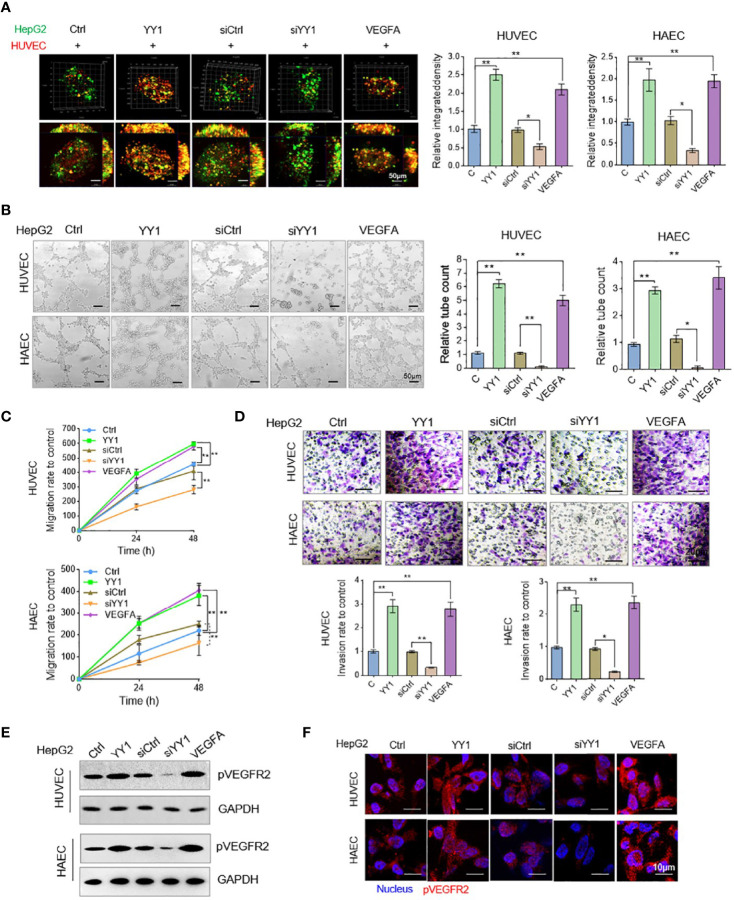
YY1 stimulated HCC cell culture media accelerated endothelial cells neovascularization. **(A)** HUVECs and HAECs (red) and HepG2 cells (green) co-cultured in a 1:2 ratio and formed three-dimensional spheroids. Images were taken with a laser scanning confocal microscope, scale bar = 50 μm. **(B)** Representative image (left) of the formation of HUVECs and HAECs tubes following an incubation with supernatants collected from the indicated cells. Tube formation quantification were analyzed (right). Scale bar = 50 μm. **(C)** HUVECs and HAECs migration were detected after an incubation with supernatants collected from the indicated cells. **(D)** HUVECs and HAECs invasion were detected following an incubation with supernatants collected from the indicated cells. Scale bar = 20 μm. **(E)** WB analyzed pVEGFR2 expression in HUVECs and HAECs treated with conditioned media. **(F)** Immunofluorescence of pVEGFR2 expression in HUVECs and HAECs treated with conditioned media. Scale bar = 10 μm. *P < 0.05, **P < 0.01.

The authors apologize for this error and state that this does not change the scientific conclusions of the article in any way. The original article has been updated.

## Publisher’s Note

All claims expressed in this article are solely those of the authors and do not necessarily represent those of their affiliated organizations, or those of the publisher, the editors and the reviewers. Any product that may be evaluated in this article, or claim that may be made by its manufacturer, is not guaranteed or endorsed by the publisher.

