# Supplementary material for: YY1 Promotes Endothelial Cell-Dependent Tumor Angiogenesis in Hepatocellular Carcinoma by Transcriptionally Activating VEGFA
Source: Front Oncol. 2019 Nov 14;9:1187. doi: 10.3389/fonc.2019.01187 (PMC6868052; doi:10.3389/fonc.2019.01187)
Supplement: Supplementary file 1 [file Data_Sheet_1.pdf]

Supplementary data

Supplementary Figure 1
Uncropped images related to Figure 3C

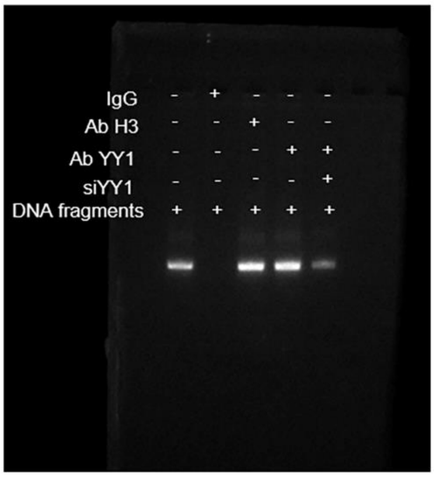

Figure S1. Uncropped bands related to Figure 3C.

Supplementary Figure 2
Uncropped blots related to Figure 3E

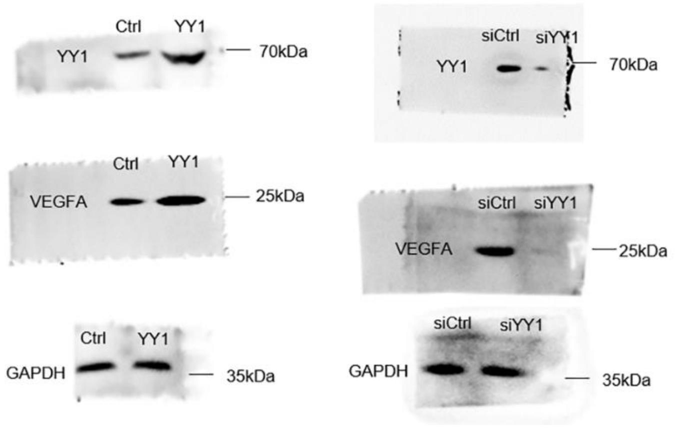

Figure S2. Uncropped blots related to Figure 3E.

Supplementary Figure 3

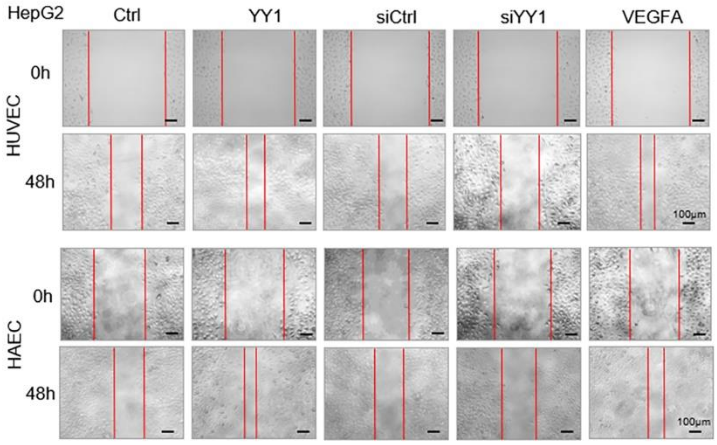

Figure S3. HUVECs and HAECs migration were detected after an incubation with supernatants collected from the indicated cells. Scale bar=100μm.

Supplementary Figure 4
Uncropped blots related to Figure 4E

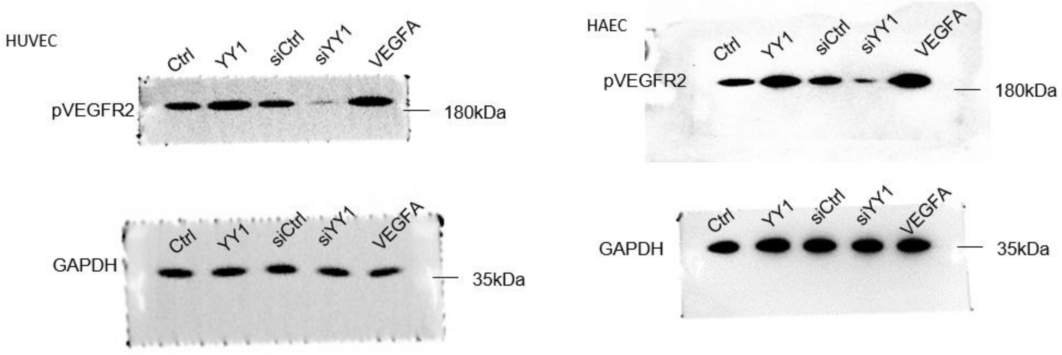

Figure S4. Uncropped blots related to Figure 4E.

Supplementary Figure 5
Uncropped blots related to Figure 5D

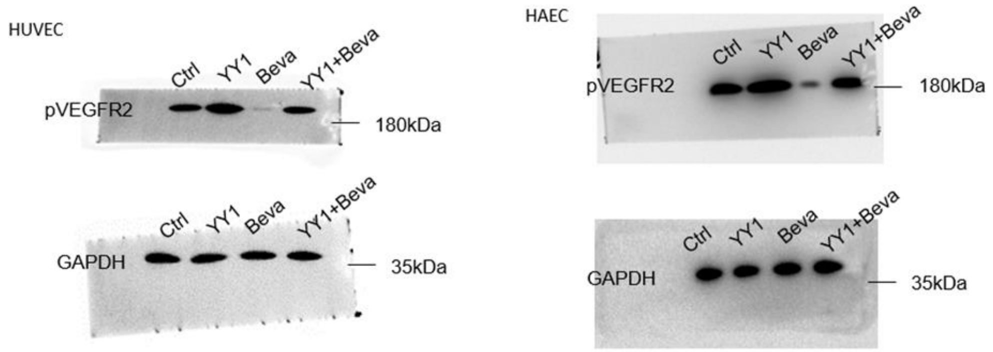

Figure S5. Uncropped blots related to Figure 5D.
